# Enhanced mobility and pain relief of total hip arthroplasty in post-traumatic ankylosing hip: A case report

**DOI:** 10.1016/j.ijscr.2025.111624

**Published:** 2025-07-08

**Authors:** M. Dimas Arya Candra Permana, M. Zaim Chilmi

**Affiliations:** a Department of Orthopedic and Traumatology, Faculty of Medicine, Airlangga University / Dr. Soetomo General Academic Hospital, Jl. Prof Moestopo 6-8, Airlangga, Gubeng, Surabaya, Indonesia; b Orthopaedic Hip and Knee Consultant, Department of Orthopedic and Traumatology, Faculty of Medicine, Airlangga University/ Dr. Soetomo General Academic Hospital, Jl. Prof Moestopo 6-8, Airlangga, Gubeng, Surabaya, Indonesia

**Keywords:** Ankylosed hips, Ankylosing spondylitis, Total hip arthroplasty, Case report

## Abstract

**Introduction and importance:**

Ankylosed hips pose significant challenges due to severe joint stiffness and deformities. Total hip arthroplasty (THA) is a critical intervention that offers pain relief and improved function, despite the technical difficulties associated with altered biomechanics and preoperative deformities. This abstract highlights the complexities of THA in Ankylosing Spondylitis (AS) patients, emphasizing the need for specialized surgical approaches to optimize outcomes and mitigate the higher risk of complications.

**Case presentation:**

A 20-year-old male presented with pain and severely restricted movement in the right leg, which began after a traumatic incident six years prior. Imaging studies, including plain radiographs, scanogram, and MSCT of the pelvis, revealed right hip osteoarthritis and ankylosis. The patient underwent a THA and was monitored for six months post-surgery.

**Clinical discussion:**

In AS, hip involvement can lead to significant complications, including synovitis, enthesitis, avascular necrosis, and secondary osteoarthritis. Bony ankylosis, a common outcome, often results in stiffness and limited mobility rather than pain. THA is primarily indicated to address stiffness or ankylosis rather than pain in these patients. Successful THA in AS patients significantly improves quality of life but requires careful management of both intraoperative and postoperative complications.

**Conclusion:**

Total hip arthroplasty, whether unilateral or bilateral, offers substantial benefits for patients with AS and severe hip ankylosis. However, careful management throughout the surgical process is essential to achieve successful outcomes and improve patient quality of life.

## Highlights

• Hip ankylosis occurs in 30–50% of AS patients, severely limiting mobility and quality of life
• Successful Total Hip Arthroplasty (THA) in a 20-year-old male with a history of ankylosing of the hip
• Improvement of Harris Hip Score (HHS) from 27 to 76 after a marked loss of ROM and poor quality of life
• Spinal AS complicates THA due to altered biomechanics affecting implant placement and durability
• Proper preoperative planning is essential to manage the complexities and successes of THA in ankylosed hips.

## 1 Introduction

Ankylosing hip, often associated with ankylosing spondylitis (AS), presents a significant challenge in orthopedic practice. This condition arises from chronic inflammation, leading to fibrosis and ossification of the joint capsule and surrounding structures, resulting in severe stiffness or complete ankylosis [1]. Hip involvement, occurring in 30–50 % of AS patients and often bilaterally, profoundly limits mobility and diminishes quality of life and 5 % of those need Total hip arthroplasty (THA). THA is a common intervention for ankylosed hips, but it is technically demanding due to altered biomechanics, joint deformities, and exposure difficulties. Despite these challenges, THA offers substantial functional improvement with appropriate preoperative planning [2,3].

In this case report, by following SCARE Guidelines, we presented a 20-year-old male with post-traumatic ankylosis of the right hip who underwent total hip replacement with improvement on his Harris Hip Score [4].

## 2 Case presentation

A 20-year-old male suffered pain in the right leg, with the history of a single accident that occurred six years ago in Papua, causing the patient to be unconscious at the time of the incident and not to seek medical facility, otherwise, the patient is healthy. The patient initially sought treatment from a traditional bonesetter, where only massages were administered. Unfortunately, over time, the patient's range of motion (ROM) became progressively limited. Eventually, he found himself unable to perform even basic movements of the right hip, such as flexion, extension, or external and internal rotation. This severe restriction has had a profound impact on his daily activities, rendering him almost entirely unable to perform routine tasks. No family history of similar complaint, no allergies or consuming any specific medication.

**Fig. 1:**
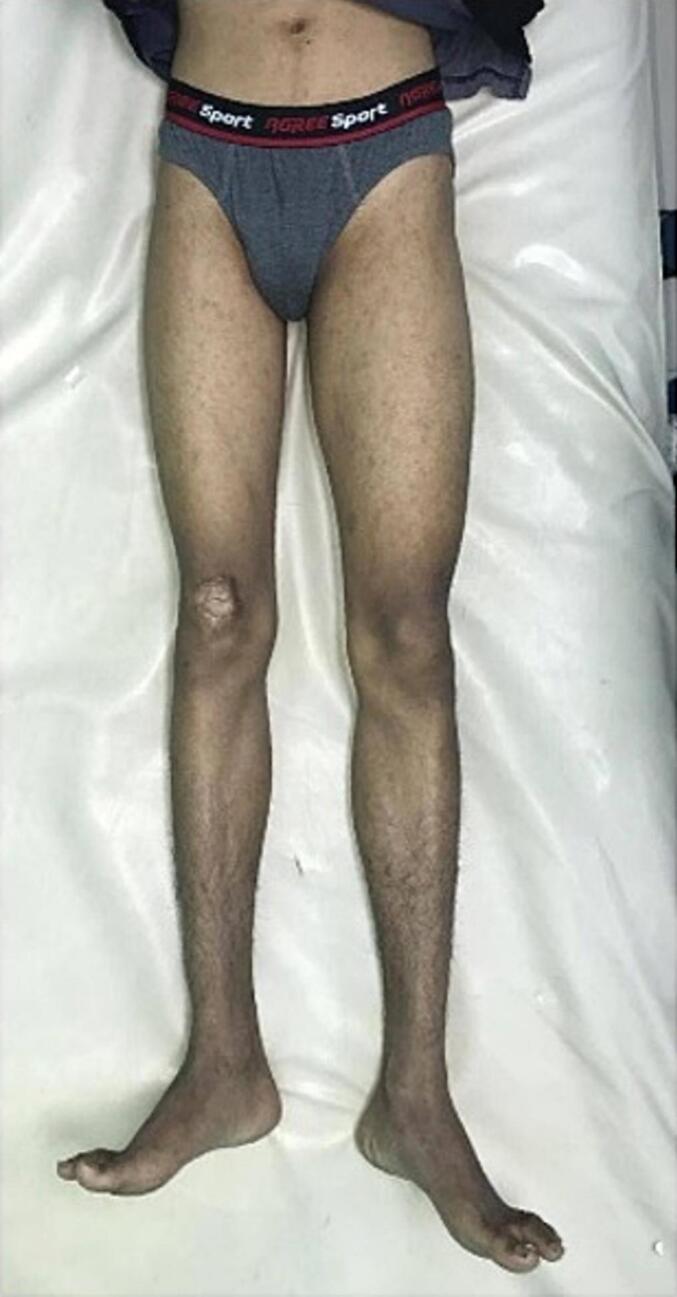
Clinical picture of leg length discrepancy (LLD).

**Fig. 2:**
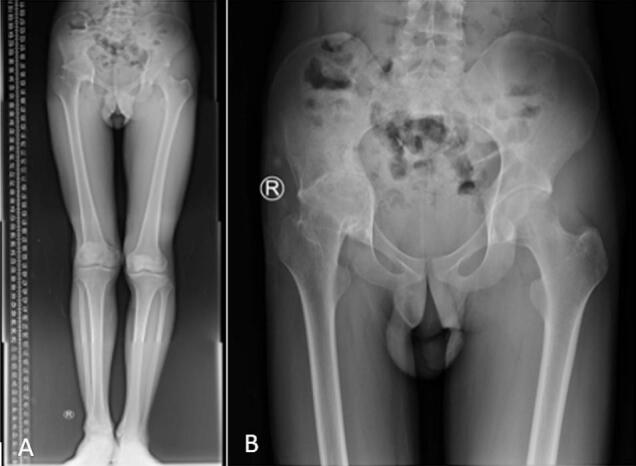
A plain radiograph of scanogram (A) and pelvic AP (B) shows ankylosing of the right hip joint.

Objectively, the general condition of the patient is sufficient. On right lower extremity, shortening deformity was found (Fig. 1). The leg length difference (LLD) is 2 cm with anatomical length 92/94 cm, tibial length 89/91 cm, and femoral length 39/39 cm. The right hip was fixed in 10^o^ flexion and 10^o^ abduction position without any active or passive range of motion. There was no tenderness or sign of inflammation. Distal joint and neurovascular status was within normal. Harris Hip Score was evaluated and scored 27 (Fig. 2).

**Fig. 3:**
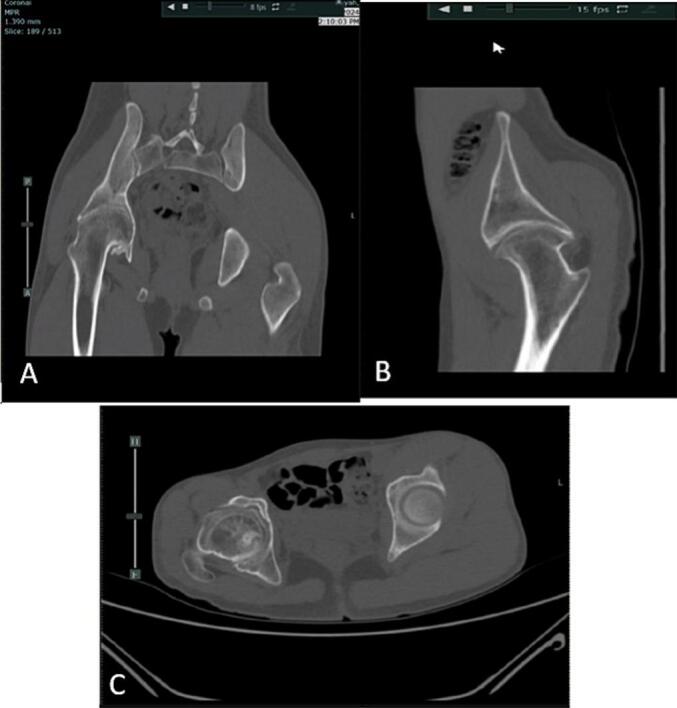
MSCT of the pelvis shows right hip osteoarthritis A. Coronal B. Sagittal C. Axial view.

The patient presented with normal laboratory test results. However, imaging studies, including a plain radiograph and scanogram, revealed secondary grade 4 osteoarthritis of the right hip joint. Further evaluation with an MSCT of the pelvis confirmed the diagnosis of right hip osteoarthritis grade IV (Fig. 3). The patient was subsequently diagnosed with post-traumatic ankylosis of the right hip and was advised to undergo total hip replacement surgery.

**Fig. 4:**
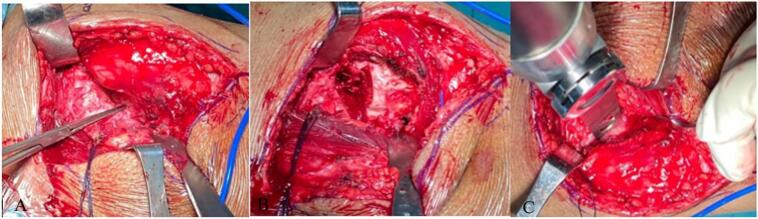
Clinical state during surgery.

The surgery was done with posterior approach (Fig. 4). Osteotomy of the neck of the femur was taken 1 cm proximal to the lesser trochanter followed by reaming the acetabulum. A 42 mm cementless acetabular cup was inserted, accompanied by the placement of two screws. A reaming procedure was performed on the intramedullary femur, followed by the insertion of a typical cementless femoral stem (size 9) together with a femoral head (size 24 mm neutral).

**Fig. 5:**
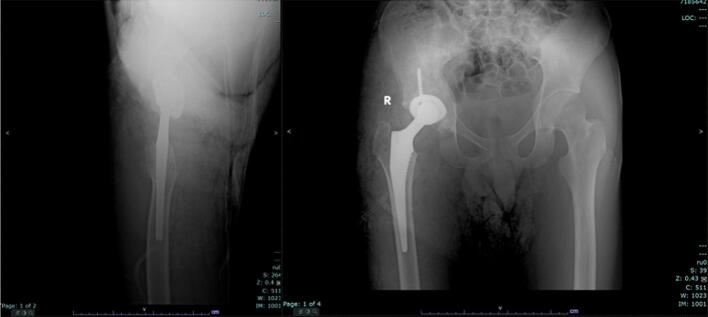
Post-operative plain radiograph of pelvic AP/Lat view.

Post-surgical radiographic assessments were done (Fig. 5) and the patient has been closely monitored over a six-month period. During this time, the patient experienced significant pain relief, with noticeable improvements in range of motion. These advancements have enabled the patient to walk more comfortably and perform daily activities with greater ease than before the procedure. At the six-month follow-up, the patient's post-operative Harris Hip Score was assessed, showing a score of 76, indicating a marked improvement in hip function, but still having a hard time to squat and sitting cross-legged.

## 3 Discussion

Ankylosing hip, commonly associated with ankylosing spondylitis (AS), is a debilitating condition characterized by fusion and stiffness of the hip joint due to chronic inflammation. Hip involvement occurs in 25–50 % of AS patients, typically bilaterally, significantly impacting mobility and quality of life. The fusion results from fibrosis and ossification of periarticular structures, leading to severe pain, stiffness, and immobility [3,5]. Non-operative management outcomes such as Disease-Modifying Antirheumatic Drugs (DMARDs), Nonsteroid Anti-Inflammatory Drugs (NSAIDs), and TNF-blockers remain unclear for ankylosing hip [6].

Total hip arthroplasty (THA) is the primary surgical intervention for these patients, aimed at restoring mobility and alleviating pain. However, performing THA in ankylosed hips is challenging due to altered anatomy, rigid bones, and structural changes [3,5].

Surgical challenges include managing osteophytes, preoperative deformities, and altered hip-spine biomechanics. Deciding between cemented and uncemented implants is critical. Cemented implants provide immediate stability, especially in patients with poor bone quality, while uncemented implants are preferred for younger patients due to their potential longevity. Proper preoperative planning is essential to address these challenges and optimize outcomes [1,7].

Case studies highlight the complexities and successes of THA in ankylosed hips. For example, a 20-year-old patient with post-traumatic hip ankylosis achieved significant pain relief and improved function after THA, with a Harris Hip Score of 76 within six months. Another case involved a 45-year-old man with AS and bilateral hip ankylosis, who experienced substantial mobility restoration following tailored THA. However, complications such as periprosthetic fractures and reankylosis can occur, requiring careful postoperative management and, in some cases, revision surgeries [8,9].

Patient-specific factors, including bone quality, severity of AS, and systemic inflammation, heavily influence surgical outcomes. Spinal involvement in AS further complicates the procedure and postoperative care, as altered biomechanics can affect implant positioning and longevity. A multidisciplinary approach involving rheumatologists, orthopedic surgeons, and rehabilitation specialists is crucial to optimize outcomes and minimize complications [10,11].

Long-term considerations include the durability of implants, particularly in younger patients, and the risk of reankylosis and implant wear. Continued research and refinement of surgical techniques are necessary to enhance the success of THA in ankylosed hips. Despite these challenges, THA remains a vital procedure, offering significant pain relief and improved quality of life for patients with ankylosing hip [12,13].

## 4 Conclusion

In conclusion, THA significantly improves mobility and quality of life for patients with ankylosing hip despite its complexity. Early intervention, tailored care, and a multidisciplinary approach are essential for success. Advances in orthopedic techniques continue to enhance outcomes, offering hope to those affected by this debilitating condition.

## Consent

Written informed consent was obtained from the patient for publication and any accompanying images. A copy of the written consent is available for review by the Editor-in-Chief of this journal on request.

## Ethical approval

The study is exempt from ethnical approval.

Ethics Committee: Komite Etik Penelitian Kesehatan/Health Research Ethics Committee Dr. Soetomo General Hospital

Ref. No: 1938/LOE/301.4.2/III/2025

## Guarantor

M. Zaim Chilmi is the sole guarantor of this submitted article.

## Research registration number

Not required.

## Disclaimer

No benefits in any forms received form the publications of this article.

## Provenance and peer review

Not commissioned, externally peer reviewed.

## Declaration of Generative AI and AI-assisted technologies in the writing process

The authors have declared no AI was used in the making of this case report.

## Funding

This research did not receive any specific grant from funding agencies in the public, commercial, or not-for-profit sectors.

## Author contribution

DAC: Data Collection, Data analysis, Writing the paper.

MZC: Study Design, Data collection, Data analysis, Editing the paper.

## Conflict of interest statement

The authors have declared no conflict of interest.
